# Supplementary figures and images for: Analysis of High-Risk Neuroblastoma Transcriptome Reveals Gene Co-Expression Signatures and Functional Features
Source: Biology (Basel). 2023 Sep 12;12(9):1230. doi: 10.3390/biology12091230 (PMC10525871; doi:10.3390/biology12091230)

## Scores

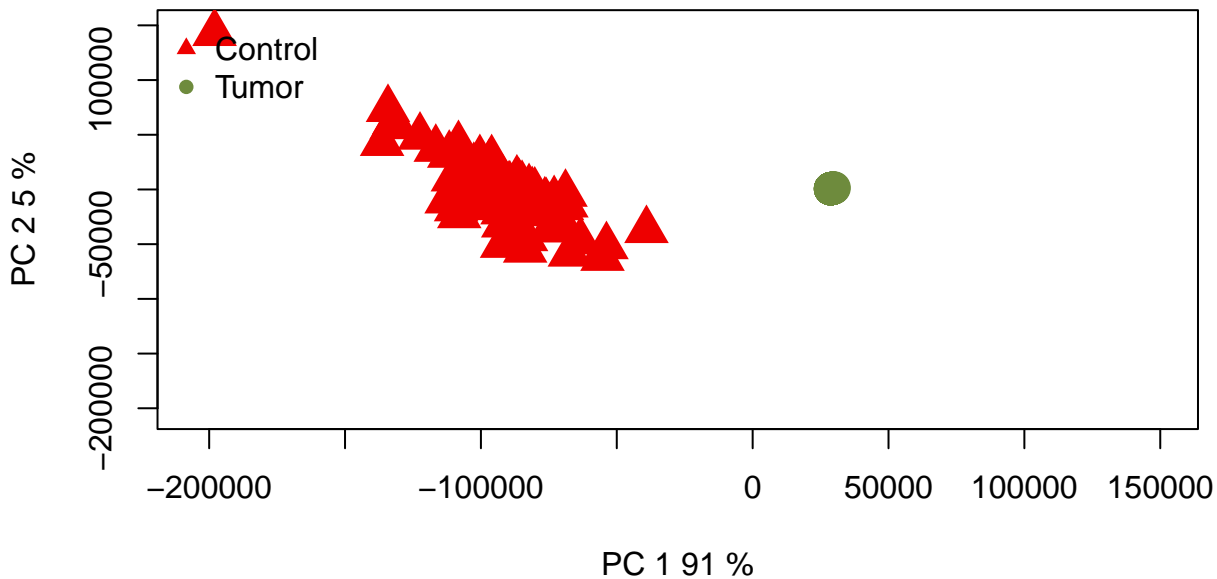

Supplement: Supplementary file 1 [file biology-12-01230-s001.zip › File S1/GSE49711.GEO_ degs/postArsyn_GSE49711.pdf]

## Scores

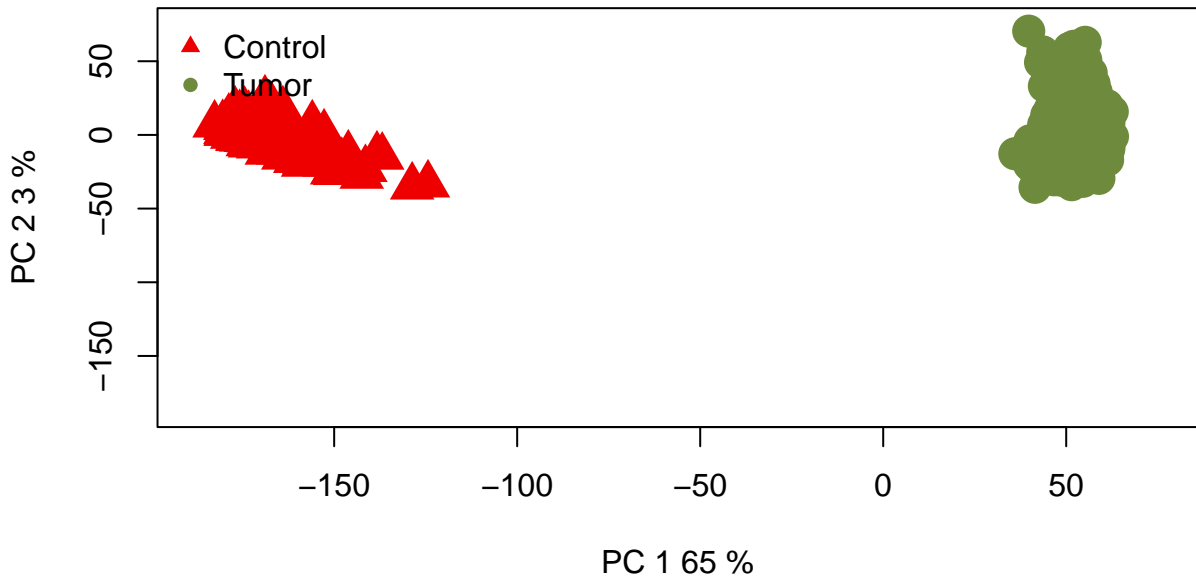

Supplement: Supplementary file 1 [file biology-12-01230-s001.zip › File S1/GSE49711.GEO_ degs/preArsyn_GSE49711.pdf]

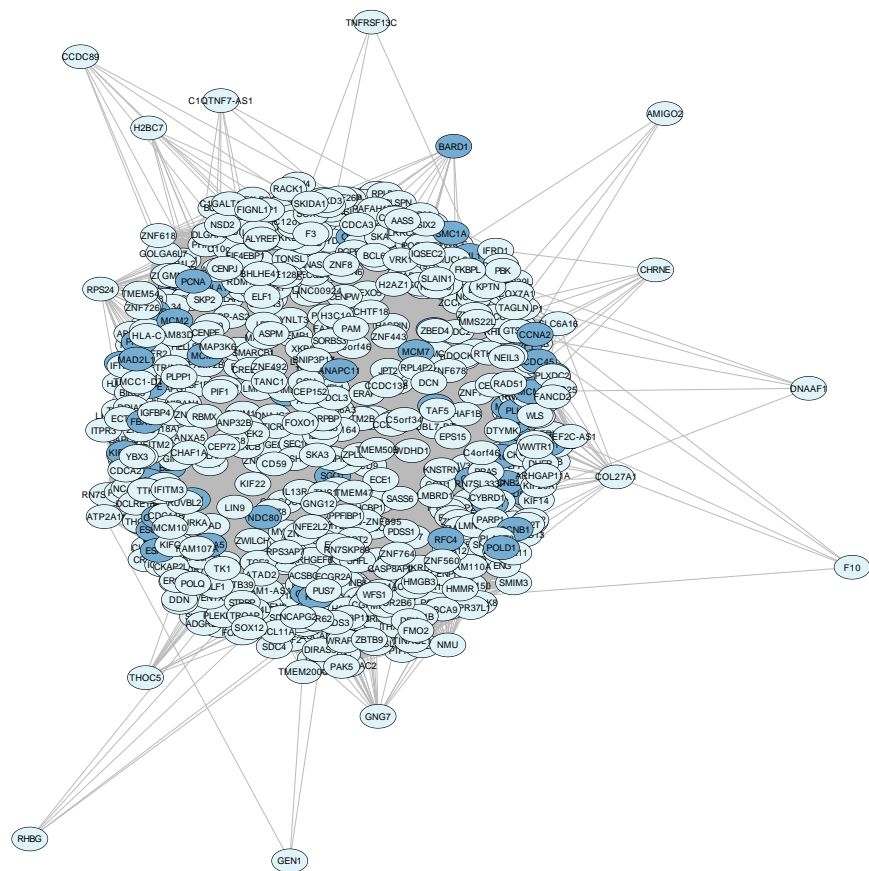

Supplement: Supplementary file 1 [file biology-12-01230-s001.zip › File S1/network_blue.pdf]

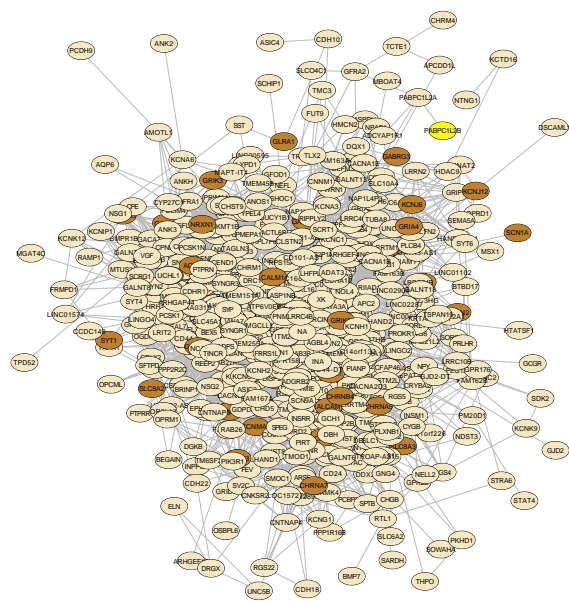

Supplement: Supplementary file 1 [file biology-12-01230-s001.zip › File S1/network_brown.pdf]

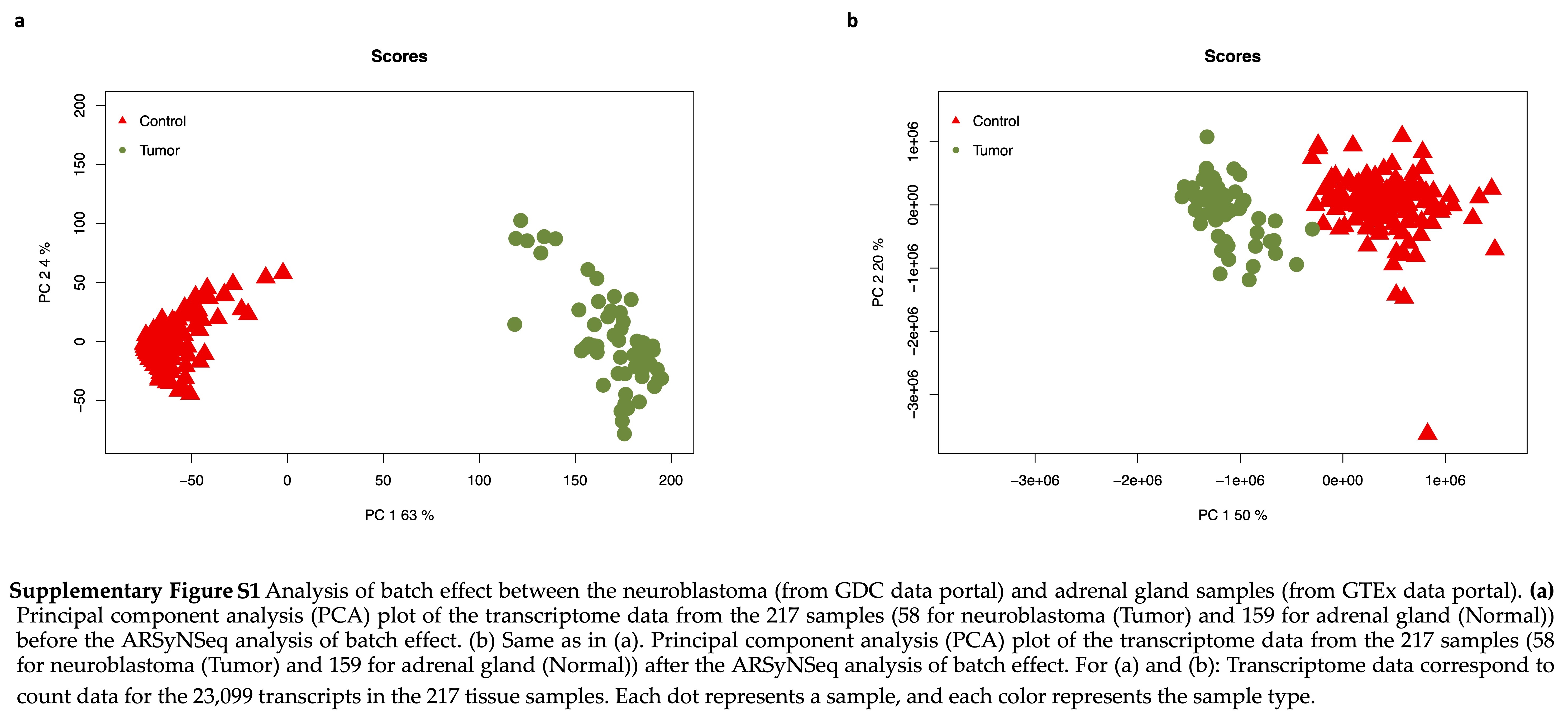

Supplement: Supplementary file 1 [file biology-12-01230-s001.zip › supplementary_figure_S1.jpg]
